# Outcomes of simultaneous laparoscopic, hybrid, and open resection in colorectal cancer with synchronous liver metastases: a propensity score-matched study

**DOI:** 10.1038/s41598-022-12372-5

**Published:** 2022-05-25

**Authors:** Han-Ki Lim, Minjung Kim, Ji Won Park, Seung-Bum Ryoo, Kyu Joo Park, Nam-Joon Yi, Kwang-Woong Lee, Kyung-Suk Suh, Heung-Kwon Oh, Duck-Woo Kim, Sung-Bum Kang, Jai Young Cho, Dong-Woon Lee, Sung Chan Park, Jae Hwan Oh, Aesun Shin, Seung-Yong Jeong

**Affiliations:** 1 Department of Surgery, Seoul National University College of Medicine, 101, Daehak-ro Jongno-gu, Seoul, 03080 Republic of Korea; 2 Colorectal Cancer Center, Seoul National University Cancer Hospital, Seoul, Republic of Korea; 3 Cancer Research Institute, Seoul National University, Seoul, Republic of Korea; 4 Department of Surgery, Seoul National University Bundang Hospital, Seongnam, Gyeonggi Republic of Korea; 5 Center for Colorectal Cancer, Research Institute and Hospital, National Cancer Center, Goyang, Gyeonggi Republic of Korea; 6 Department of Preventive Medicine, Seoul National University College of Medicine, Seoul, Republic of Korea

**Keywords:** Gastroenterology, Oncology

## Abstract

We aimed to compare the short- and long-term outcomes of simultaneous laparoscopic, hybrid, and open resection for colorectal cancer and synchronous liver metastases. We retrospectively analyzed the data of 647 patients with simultaneous resection of colorectal cancer and liver metastases between January 2006 and December 2018 at three tertiary referral hospitals. Patient’s baseline characteristics, perioperative outcomes, pathological examination results, liver-specific recurrence rate and survivals were compared between the propensity score-matched groups. Forty-two and 81 patients were selected for the laparoscopic vs. hybrid groups, and 48 and 136 patients for laparoscopic vs. open groups, respectively. The laparoscopic group had fewer wound complications (2.1 vs. 13.2%; p = 0.028) than the open group, and a shorter postoperative hospital stay than the hybrid and open groups (8 vs. 11 days, p < 0.001 for both). The 5-year liver-specific recurrence rates were 38.7% and 46.0% in the laparoscopic and hybrid groups, respectively (p = 0.270), and 34.0% and 37.0% in the laparoscopic and open groups, respectively (p = 0.391). Simultaneous laparoscopic resection for colorectal cancer and liver metastases can be performed safely with significantly enhanced postoperative recovery and comparable long-term outcomes compared to hybrid and open resection.

## Introduction

The liver is the most common site of metastasis in colorectal cancer, and the survival of patients with metastatic hepatic disease is poor. Synchronous liver metastases are present in 15–25% of patients diagnosed with colorectal cancer, and metachronous liver metastases will develop in 20–30% of patients during the postoperative follow-up period^1,2^. The 3-year survival rate of patients with colorectal cancer and unresectable liver metastases has been reported to be as low as 3–9%, with a median survival of 5–10 months if left untreated^3–7^. Complete resection of both the primary colorectal cancer and liver metastases improves the 5-year survival rate by 40–57%^8,9^.

Major liver resection is associated with a significant risk of fatal postoperative complications, such as pulmonary (20%) and cardiovascular (9%) complications, liver failure (3–8%), bile leak (4%), perihepatic abscess (2–10%), and hemorrhage (1–3%)^10–15^. Surgeons need to be highly experienced to achieve oncologically adequate resection margins. Therefore, some institutions used to perform liver resection in a staged open approach rather than as a simultaneous laparoscopic procedure.

However, this has recently changed with the continuous improvement in instruments and surgical techniques. Recent studies and meta-analyses compared simultaneous with staged resection of the primary tumor and liver metastases and concluded that simultaneous surgery is safe and achieves better short-term outcomes^16,17^. Furthermore, advanced laparoscopic techniques, such as three-dimensional (3D) laparoscopy and indocyanine green (ICG) fluorescence imaging, substantially improve depth perception and anatomical orientation. With this new technology, laparoscopic liver resection can be safely performed by experienced professionals^18,19^. Therefore, simultaneous laparoscopic resection of colorectal cancer and liver metastases is now increasingly performed, particularly at specialized centers^20,21^.

However, retrospective studies on simultaneous laparoscopic resection inevitably have a profound selection bias and remain inconclusive because more advanced liver metastases tend to be resected using an open rather than laparoscopic approach^22–25^. Furthermore, the surgical outcomes of hybrid, laparoscopic, and open colorectal cancer and liver resection have rarely been evaluated separately, and data on long-term survival after laparoscopic liver resection are missing.

Consequently, this study aimed to compare the short-term and long-term outcomes in patients undergoing simultaneous laparoscopic, hybrid, and open resection of colorectal cancer and liver metastases using propensity score matching.

## Methods

### Study design

This was a retrospective study reviewing prospectively collected data in a multicenter database. The study protocol was approved by the Institutional Review Board of the Seoul National University Hospital (institutional review board No. 2104-227-1217). All procedures of this study were performed in accordance with relevant guidelines and regulations of institutional review board, and complied with the ethical principles in the Declaration of Helsinki. The institutional review board waived the need for informed consent because of the nature of the study as a retrospective file review.

### Patients

A total of 675 patients had undergone simultaneous surgery for primary colorectal cancer and synchronous liver metastases with curative intent between January 2006 and December 2018 at three tertiary referral hospitals, performing > 700 laparoscopic colorectal surgeries and > 300 laparoscopic liver resections annually, were eligible for this study. Six hundred and sixty-eight patients with histologically proven synchronous colorectal adenocarcinoma and liver metastases, without a history of other malignancies, were included. Patients who had double primary colorectal cancer (10 patients), had undergone an incomplete resection of either the primary tumor, the liver metastases, or both (5 patients), had concomitant extrahepatic metastatic disease (2 patients), or were treated only with radiofrequency ablation for liver metastases (4 patients) were excluded. As a result, 647 patients were enrolled in this study.

Data on patients’ baseline characteristics, perioperative outcomes, pathological examination results, recurrence, and survival were collected.

### Preoperative workup

All patients underwent physical examination, colonoscopy, and computed tomography (CT) scanning of the chest, abdomen, and pelvis with or without magnetic resonance imaging (MRI) scans of the liver before surgery to evaluate the preoperative stage. Whole-body positron emission tomography-CT scans were performed in selected cases depending on the extent of the disease.

### Surgical procedure

All patients with simultaneous colorectal and liver resection could be classified into three groups according to their surgical treatment: laparoscopic, hybrid, and open.

In the laparoscopic group, both colorectal resection and hepatectomy were performed laparoscopically by experienced laparoscopic surgeons. In the open group, patients underwent both colorectal and liver resection during laparotomy procedures. In the hybrid group, laparoscopic colorectal resection was followed by open liver resection.

All simultaneous colorectal and liver resections were performed by two separate surgical teams. Laparoscopic colorectal resection was performed using the conventional five-port method. Laparoscopic ultrasonography was used to assess the resectability of the metastases and guide liver resection. Since 2016, we have utilized tumor visualization with ICG fluorescence in the PINPOINT near-infrared imaging system (Stryker Corporation, Kalamazoo, MI, USA) in cases where the localization of liver metastases was difficult or in cases where liver cirrhosis was severe. In major laparoscopic liver resection, we used real-time intraoperative liver mapping using ICG fluorescence to improve visualization of the hepatobiliary anatomy^19^. Transection of the liver was performed using an ultrasound dissector and/or ultrasonic surgical aspirator to ensure adequate margins around the metastases. The hepatic vein was divided using a linear stapler. The resected liver specimen was extracted in a plastic bag through the mini-laparotomy at the extended umbilical port site, which had been used to extract the colorectal specimen.

For open colorectal and liver resections, we performed laparotomy using a midline incision. If necessary, an additional transverse incision was made for open hepatectomy.

Major liver resection was defined as the resection of three or more adjacent segments, and minor liver resection was defined as the resection of fewer than three segments.

### Pathological examination findings

The surgical specimens of the primary tumor and liver metastases were evaluated by board-certified pathologists, and the pathologic stage was determined based on the eighth edition of the American Joint Committee on Cancer Staging System^26^. The maximal size of liver metastases was documented as the largest diameter of the lesion on gross examination of the specimen. The hepatic resection margin was considered positive if the distance from the tumor to the surgical resection margin was less than 1 mm microscopically^27^.

### Postoperative and oncologic outcomes

Postoperative outcomes, including morbidity and mortality within 90 days of surgery, were evaluated. The severity of complications was evaluated according to the modified Clavien-Dindo classification^28^. All cases requiring procedures other than the routine stitch out on postoperative day 7 were included in wound complications. Patients were followed up every 3 months for the first 2 years after surgery, then every 6 months for up to 5 years, and once a year thereafter. Recurrence was demonstrated by pathological results obtained by surgical resection, biopsy, or cytology of the recurrent mass and/or radiological findings of an increase in tumor size over time. Liver-specific recurrence was defined as the detection of tumor recurrence within the liver parenchyma.

### Statistical analysis

Propensity score matching was performed using the nearest neighbor matching with a caliper of 0.2 to reduce a potential imbalance of the covariates between the three groups. The matching considered seven variables that might influence the perioperative and oncological outcomes: the primary tumor location, p/ypT stage, the number and distribution of liver metastases, postero-superior segments or caudate lobe involvement, close proximity to major vessels, and type of liver resection. After propensity score matching, the laparoscopic group was matched at a ratio of 1:3 with the hybrid and open groups, respectively.

The characteristics of patients in the laparoscopic, hybrid, and open groups were compared using the Student's t-test or Mann–Whitney U test for continuous variables and the chi-square test or Fisher's exact test for categorical variables. Survival curves were estimated using the Kaplan–Meier method, and curves were compared with the log-rank test.

In our calculation of the cumulative liver-specific recurrence rate and overall survival (OS), we defined the first liver-specific relapse and death by any cause after complete resection as an event. In our determination of disease-free survival (DFS), any local or metastatic recurrence, either hepatic or extrahepatic, second primary colorectal cancers, or death of any cause after complete resection was defined as an event.

Statistical significance was defined as a p value < 0.05. All statistical analyses were performed using IBM SPSS Statistics for Windows, version 25 (IBM Corp., Armonk, NY, USA).

## Results

A total of 647 patients (mean age 60.0 years, 36.2% females) were analyzed, and the baseline characteristics of the study population are listed in Table 1.

**Table 1:** Patients and disease characteristics according to treatment group.

	Before propensity score matching				After propensity score matching					
	Lap (n = 51)	Hybrid (n = 154)	Open (n = 442)	p value	Lap (n = 42)	Hybrid (n = 81)	p value	Lap (n = 48)	Open (n = 136)	p value
**Patients**										
Age, median (range)	63 (23–76)	60 (31–85)	60 (21–86)	0.947	64 (23–76)	59 (31–85)	0.455	61 (23–76)	62 (21–86)	0.568
** Gender, n (%)**				0.982			0.885			0.939
Male	32 (62.7)	98 (63.6)	283 (64.0)		27 (64.3)	51 (63.0)		31 (64.6)	87 (64.0)	
Female	19 (37.3)	56 (36.4)	159 (36.0)		15 (35.7)	30 (37.0)		17 (35.4)	49 (36.0)	
** Race, n (%)**				0.628			N.A			> 0.999
Asian	51 (100)	154 (100)	440 (99.5)		42 (100)	81 (100)		48 (100)	135 (99.3)	
White	0 (0)	0 (0)	2 (0.5)		0 (0)	0 (0)		0 (0)	1 (0.7)	
BMI, median (range)	23.4 (16.8–31.8)	23.3 (17.0–32.1)	23.0 (13.9–36.1)	0.715	23.6 (16.8–31.8)	23.2 (18.3–30.1)	0.957	23.4 (16.8–31.8)	22.7 (16.6–32.4)	0.404
** ASA-PS, n (%)**				0.973			0.605			> 0.999
I–II	49 (96.1)	148 (96.1)	423 (95.7)		40 (95.2)	79 (97.5)		46 (95.8)	128 (94.1)	
III–IV	2 (3.9)	6 (3.9)	19 (4.3)		2 (4.8)	2 (2.5)		2 (4.2)	8 (5.9)	
**Colorectal tumor**										
Location, n (%)				< 0.001			0.544			0.787
Rt. colon	10 (19.6)	6 (3.9)	104 (23.5)		5 (11.9)	6 (7.4)		9 (18.8)	32 (23.5)	
Lt. colon	22(43.1)	57 (37.0)	179 (40.5)		19 (45.2)	33 (40.7)		21 (43.8)	57 (41.9)	
Rectum	19 (37.3)	91 (59.1)	159 (36.0)		18 (42.9)	42 (51.9)		18 (37.5)	47 (34.6)	
**Liver metastases**										
Number of lesions, n (%)				0.030			0.899			0.801
1	29 (56.9)	74 (48.1)	166 (37.6)		23 (54.8)	42 (51.9)		27 (56.3)	79 (58.1)	
2–4	17 (33.3)	61 (39.6)	205 (46.4)		14 (33.3)	27 (33.3)		16 (33.3)	47 (34.6)	
> 4	5 (9.8)	19 (12.3)	71 (16.1)		5 (11.9)	12 (14.8)		5 (10.4)	10 (7.4)	
** Distribution, n (%)**				0.020			0.320			0.790
Unilobar	42 (82.4)	103 (66.9)	278 (62.9)		34 (81.0)	59 (72.8)		40 (83.3)	111 (81.6)	
Bilobar	9 (17.6)	51 (33.1)	164 (37.1)		8 (19.0)	22 (27.2)		8 (16.7)	25 (18.4)	
** Location, n (%)**										
Segment 1	0 (0)	10 (6.5)	20 (4.5)	0.158	0 (0)	4 (4.9)	0.298	0 (0)	3 (2.2)	0.569
Segment 2	9 (17.6)	34 (22.1)	93 (21.0)	0.797	8 (19.0)	18 (22.2)	0.683	9 (18.8)	21 (15.4)	0.594
Segment 3	12 (23.5)	25 (16.2)	82 (18.6)	0.501	9 (21.4)	12 (14.8)	0.355	11 (22.9)	20 (14.7)	0.191
Segment 4a	9 (17.6)	30 (19.5)	94 (21.3)	0.775	9 (21.4)	16 (19.8)	0.827	9 (18.8)	14 (10.3)	0.128
Segment 4b	4 (7.8)	12 (7.8)	49 (11.1)	0.434	3 (7.1)	9 (11.1)	0.750	4 (8.3)	12 (8.8)	> 0.999
Segment 5	15 (29.4)	39 (25.3)	126 (28.5)	0.724	12 (28.6)	20 (24.7)	0.642	15 (31.3)	35 (25.7)	0.460
Segment 6	16 (31.4)	59 (38.3)	173 (39.1)	0.558	13 (31.0)	32 (39.5)	0.350	14 (29.2)	45 (33.1)	0.617
Segment 7	12 (23.5)	45 (29.2)	165 (37.3)	0.045	11 (26.2)	20 (24.7)	0.856	11 (22.9)	36 (26.5)	0.627
Segment 8	12 (23.5)	47 (30.5)	155 (35.1)	0.188	12 (28.6)	21 (25.9)	0.754	12 (25.0)	33 (24.3)	0.919
Postero-superior segments or caudate lobe*, n (%)	26 (51.0)	101 (65.6)	311 (70.4)	0.016	25 (59.5)	48 (59.3)	0.977	25 (52.1)	71 (52.2)	0.988
Close proximity to major vessels^†^, n (%)	2 (3.9)	8 (5.2)	30 (6.8)	0.617	2 (4.8)	4 (4.9)	> 0.999	2 (4.2)	9 (6.6)	0.731
Diaphragm infiltration	0 (0)	0 (0)	0 (0)	N.A	0 (0)	0 (0)	N.A	0 (0)	0 (0)	N.A
** Neoadjuvant Tx, n (%)**				0.035			0.040			0.348
No	46 (90.2)	112 (72.7)	351 (79.4)		38 (90.5)	62 (76.5)		43 (89.6)	115 (84.6)	
CTx only	1 (2.0)	27 (17.5)	64 (14.5)		1 (2.4)	15 (18.5)		1 (2.1)	11 (8.1)	
CRTx	4 (7.8)	15 (9.7)	27 (6.1)		3 (7.1)	4 (4.9)		4 (8.3)	10 (7.4)	
** CEA level, median (range)**				0.058			0.237			0.576
≤ 5 ng/mL	21 (43.8)	41 (26.8)	148 (34.8)		17 (43.6)	26 (32.5)		20 (44.4)	52 (39.7)	
> 5 ng/mL	27 (56.3)	112 (73.2)	277 (65.2)		22 (56.4)	54 (67.5)		25 (55.6)	79 (60.3)	

Lap, laparoscopic; BMI, body mass index; ASA-PS, American Society of Anesthesiologists physical status classification, Rt. colon, right colon; Lt. colon, left colon; Tx, therapy; CTx, chemotherapy; CRTx, chemoradiotherapy; CEA, carcinoembryonic antigen; N.A., not applicable.

*Segments 1, 4a, 7, and/or 8.

^†^The hilum, major hepatic veins, and/or inferior vena cava.

The median follow-up time in the entire study population was 58.6 months (range 0.8–186.4). The median duration of follow-up for all patients in the matched laparoscopic and hybrid groups was 69.3 months (range 5.3–178.7) and 59.7 months (range 2.6–181.8) in the matched laparoscopic and open groups.

Significantly more cases of locally advanced colorectal cancer (p/ypT3–4) were observed in the open group (96.8%) and hybrid group (94.8%) than in the laparoscopic group (84.3%, p < 0.001). We also found significantly more multiple liver metastases in the open (2–4 nodules 46.4%, > 4 nodules 16.1%) and hybrid groups (2–4 nodules 39.6%, > 4 nodules 12.3%) than in the laparoscopic group (2–4 nodules 33.3%, > 4 nodules 9.8%, p = 0.030). The same applied for bilobar metastases (open group 37.1%, hybrid group 33.1%, laparoscopic group 17.6%, p = 0.020) and postero-superior segments or caudate lobe involvement (open group 70.4%, hybrid group 65.6%, laparoscopic group 51.0%, p = 0.016). The proportions close to major vessels were comparable between the three groups (open group 3.9%, hybrid group 5.2%, laparoscopic group 6.8%, p = 0.617) and there was no metastatic infiltration into the diaphragm.

**Figure 1:**
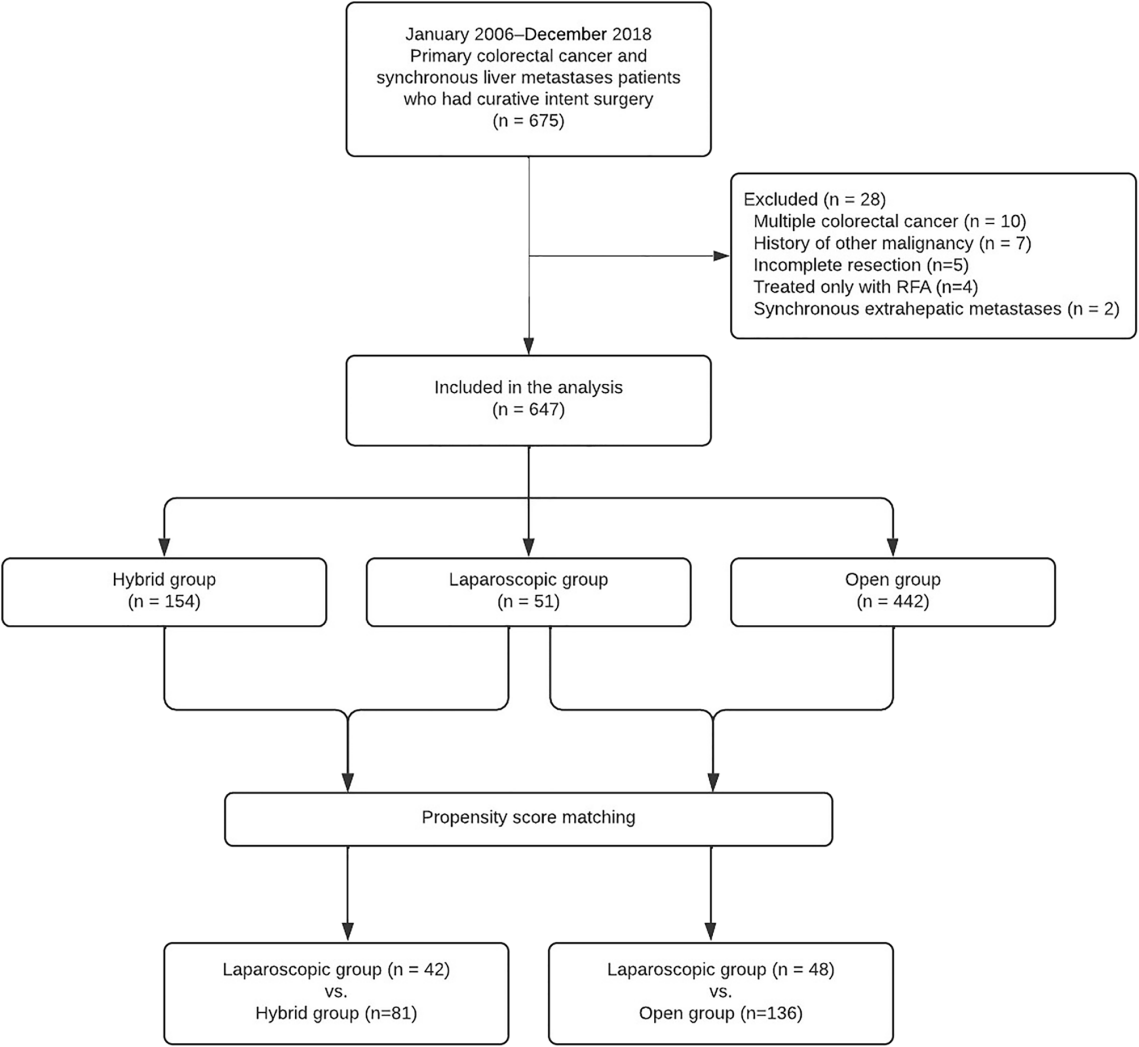
Flowchart of the study population. RFA, radiofrequency ablation.

After propensity score matching, 42 and 81 patients were included in the laparoscopic and hybrid groups, respectively, for the comparison between the two approaches, and 48 and 136 patients were selected for the laparoscopic and open groups, respectively, to compare these two approaches (Fig. 1). The baseline characteristics were comparable between the matched groups, except for the ratio of neoadjuvant treatment between the laparoscopic and hybrid groups (Table 1).

The operative data of the colorectal tumor resection were not statistically significantly different between the matched groups (Table 2). Major liver resection was performed in 19.0% vs. 21.0% in the laparoscopic vs. hybrid group (p = 0.800) and 16.7% vs. 15.4% in the laparoscopic vs. open group (p = 0.841). The conversion rate from laparoscopic to open liver resection was 7.7% (n = 4) in the laparoscopic group before matching. The reasons for conversion to open surgery were major vessel injury (1), difficulty in approach (1), and surgical complexity because of multiple lesions (2).

**Table 2:** Surgical characteristics according to treatment group.

	Before propensity score matching				After propensity score matching					
	Lap (n = 51)	Hybrid (n = 154)	Open (n = 442)	p value	Lap (n = 42)	Hybrid (n = 81)	p value	Lap (n = 48)	Open (n = 136)	p value
**Colorectal tumor**										
Colorectal resection, n (%)				< 0.001			0.589			0.851
Right-sided colectomy (RHC)	10 (19.6)	6 (3.9)	93 (21.0)		5 (11.9)	6 (7.4)		9 (18.8)	29 (21.3)	
Left-sided colectomy (LHC/AR)	16 (31.4)	55 (35.7)	149 (33.7)		13 (31.0)	32 (39.5)		15 (31.3)	45 (33.1)	
Rectal resection (LAR/ULAR/ISR/Hartmann)	22 (43.1)	87 (56.5)	168 (38.0)		21 (50.0)	41 (50.6)		21 (43.8)	53 (39.0)	
APR	2 (3.9)	5 (3.2)	11 (2.5)		2 (4.8)	1 (1.2)		2 (4.2)	3 (2.2)	
STC/TC/TPC	1 (2.0)	1 (0.6)	21 (4.8)		1 (2.4)	1 (1.2)		1 (2.1)	6 (4.4)	
Stoma, n (%)	12 (23.5)	53 (34.4)	94 (21.3)	0.005	11 (26.2)	24 (29.6)	0.689	12 (25.0)	32 (23.5)	0.837
**Liver metastases**										
Extent of liver resection, n (%)				0.008			0.800			0.841
Minor	43 (84.3)	96 (62.3)	277 (62.7)		34 (81.0)	64 (79.0)		40 (83.3)	115 (84.6)	
Major	8 (15.7)	58 (37.7)	165 (37.3)		8 (19.0)	17 (21.0)		8 (16.7)	21 (15.4)	
**Liver resection, n (%)**				< 0.001			0.302			0.391
Tumorectomy	31 (60.8)	41 (26.6)	168 (38.0)		23 (54.8)	37 (45.7)		28 (58.3)	84 (61.8)	
Segmentectomy	7 (13.7)	17 (11.0)	43 (9.7)		6 (14.3)	10 (12.3)		7 (14.6)	11 (8.1)	
Sectionectomy	5 (9.8)	42 (27.3)	73 (16.5)		5 (11.9)	20 (24.7)		5 (10.4)	22 (16.2)	
Hemihepatectomy	8 (15.7)	45 (29.2)	127 (28.7)		8 (19.0)	11 (13.6)		8 (16.7)	16 (11.8)	
Trisectionectomy	0 (0)	9 (5.8)	31 (7.0)		0 (0)	3 (3.7)		0 (0)	3 (2.2)	
Conversion, n (%)	4 (7.7)	N.A	N.A	N.A	4 (9.5)	N.A	N.A	4 (8.3)	N.A	N.A

Lap, laparoscopic; RHC, right hemicolectomy; LHC, left hemicolectomy; AR, anterior resection; LAR, low anterior resection; ULAR, ultralow anterior resection; ISR, intersphincteric resection; APR, abdominoperineal resection; STC, subtotal colectomy; TC, total colectomy; TPC, total proctocolectomy; N.A., not applicable.

In terms of perioperative outcomes in the matched groups, the laparoscopic group showed a shorter postoperative hospital stay than the other groups (Table 3). Operative time, estimated blood loss, transfusion rate, and pathologic outcomes, including p/ypT stage, p/ypN stage, differentiation of primary colorectal cancer, and the maximal lesion size and positive resection margin rate of liver metastases were comparable between the matched groups. The laparoscopic group showed similar overall morbidity rates (28.6% vs. 25.9%, p = 0.754) and Clavien–Dindo classification (Grade I and II 16.7% vs. 18.5%; Grade III and IV 11.9% vs. 7.4%, p = 0.703), but significantly higher postoperative biliary collections (7.1% vs. 0%, p = 0.038) compared to the hybrid group (Table 3). Compared to the open group, the laparoscopic group also showed similar overall morbidity rates (25.0% vs. 33.8%, p = 0.258) and Clavien-Dindo classification (Grade I and II 14.6% vs. 21.3%; Grade III and IV 10.4% vs. 12.5%, p = 0.508), but statistically significantly lower wound complications (2.1% vs. 13.2%, p = 0.028). There were six (1.4%) 90-day postoperative mortality in the open group before matching. The reasons for mortality were hepatic failure (2), septic shock due to anastomotic leakage (1) and biliary collection (1), pneumonia (1), and rapid cancer progression (1).

**Table 3:** Intra- and postoperative outcomes according to treatment group.

	Before propensity score matching				After propensity score matching					
	Lap (n = 51)	Hybrid (n = 154)	Open (n = 442)	p value	Lap (n = 42)	Hybrid (n = 81)	p value	Lap (n = 48)	Open (n = 136)	p value
Operating time (min), median (range)	300 (162–670)	363 (90–924)	316 (60–725)	0.001	315 (162–670)	330 (90–679)	0.707	308 (162–670)	279 (82–725)	0.070
EBL (mL), median (range)	300 (20–1500)	400 (50–1500)	400 (10–14,500)	< 0.001	325 (20–1500)	300 (50–1400)	0.516	300 (20–1500)	350 (30–14,500)	0.063
Transfusion, n (%)	5 (9.8)	12 (7.8)	63 (14.3)	0.094	4 (9.5)	5 (6.2)	0.489	5 (10.4)	19 (14.0)	0.530
**Colorectal tumor**										
p/ypT stage, n (%)				< 0.001			0.508			0.158
T1–2	8 (15.7)	8 (5.2)	14 (3.2)		5 (11.9)	6 (7.4)		5 (10.4)	6 (4.4)	
T3–4	43 (84.3)	146 (94.8)	428 (96.8)		37 (88.1)	75 (92.6)		43 (89.6)	130 (95.6)	
p/ypN stage, n (%)				0.299			0.937			0.626
N0	8 (15.7)	18 (11.7)	75 (17.0)		6 (14.3)	12 (14.8)		7 (14.6)	24 (17.6)	
N1–2	43 (84.3)	136 (88.3)	367 (83.0)		36 (85.7)	69 (85.2)		41 (85.4)	112 (82.4)	
**Differentiation, n (%)**				0.271			0.307			0.224
WD/MD	45 (88.2)	146 (94.8)	412 (93.2)		37 (88.1)	76 (93.8)		42 (87.5)	127 (93.4)	
PD/Mucinous/SRC	6 (11.8)	8 (5.2)	30 (6.8)		5 (11.9)	5 (6.2)		6 (12.5)	9 (6.6)	
** Liver metastases**										
Maximal lesion size (cm), median (range)	2.0 (0.5–7.0)	2.0 (0.3–16.1)	2.3 (0.1–15.4)	0.011	2.0 (0.5–7.0)	1.9 (0.3–16.1)	0.669	2.0 (0.5–7.0)	2.0 (0.4–12.0)	0.937
Positive RM, n (%)	5 (9.8)	27 (17.5)	63 (14.3)	0.280	5 (11.9)	12 (14.8)	0.657	5 (10.4)	11 (8.1)	0.567
Length of RM (cm), median (range)	0.40 (0–3.5)	0.50 (0–8.5)	0.40 (0–11.0)	0.194	0.4 (0–3.5)	0.6 (0–8.0)	0.252	0.4 (0–3.5)	0.5 (0–9.1)	0.442
Postop hospital stay (days), median (range)	8 (6–21)	11 (5–51)	12 (5–79)	< 0.001	8 (6–21)	11 (7–40)	< 0.001	8 (6–21)	11 (5–79)	< 0.001
Morbidity*, n (%)	12 (23.5)	48 (31.2)	170 (38.5)	0.046	12 (28.6)	21 (25.9)	0.754	12 (25.0)	46 (33.8)	0.258
Colorectal morbidity	2 (3.9)	4 (2.6)	10 (2.3)	0.765	2 (4.8)	1 (1.2)	0.268	2 (4.2)	1 (0.7)	0.167
Anastomotic leakage	2 (3.9)	4 (2.6)	5 (1.1)	0.212	2 (4.8)	1 (1.2)	0.268	2 (4.2)	1 (0.7)	0.167
Intestinal ischemia	0 (0)	0 (0)	2 (0.5)	0.628	0 (0)	0 (0)	N.A	0 (0)	0 (0)	N.A
Intestinal stricture	0 (0)	0 (0)	2 (0.5)	0.628	0 (0)	0 (0)	N.A	0 (0)	0 (0)	N.A
Anastomotic bleeding	0 (0)	0 (0)	1 (0.2)	0.793	0 (0)	0 (0)	N.A	0 (0)	0 (0)	N.A
Liver morbidity	3 (5.9)	3 (1.9)	17 (3.8)	0.354	3 (7.1)	1 (1.2)	0.115	3 (6.3)	3 (2.2)	0.184
Biliary collection	3 (5.9)	1 (0.6)	13 (2.9)	0.098	3 (7.1)	0 (0)	0.038	3 (6.3)	3 (2.2)	0.184
Biliary obstruction	0 (0)	0 (0)	1 (0.2)	0.793	0 (0)	0 (0)	N.A	0 (0)	0 (0)	N.A
Hepatic bleeding	0 (0)	2 (1.3)	0 (0)	0.040	0 (0)	1 (1.2)	> 0.999	0 (0)	0 (0)	N.A
Hepatic failure	0 (0)	0 (0)	3 (0.7)	0.497	0 (0)	0 (0)	N.A	0 (0)	0 (0)	N.A
Portal vein thrombosis	0 (0)	0 (0)	2 (0.5)	0.628	0 (0)	0 (0)	N.A	0 (0)	0 (0)	N.A
General morbidity	7 (13.7)	44 (28.6)	144 (32.6)	0.019	7 (16.7)	19 (23.5)	0.382	7 (14.6)	43 (31.6)	0.023
Wound Cx	1 (2.0)	14 (9.1)	70 (15.8)	0.005	1 (2.4)	8 (9.9)	0.164	1 (2.1)	18 (13.2)	0.028
Prolonged ileus	1 (2.0)	13 (8.4)	32 (7.2)	0.291	1 (2.4)	5 (6.2)	0.663	1 (2.1)	10 (7.4)	0.293
Urinary retention	3 (5.9)	16 (10.4)	20 (4.5)	0.031	3 (7.1)	6 (7.4)	> 0.999	3 (6.3)	7 (5.1)	0.722
Cardiovascular Cx	0 (0)	0 (0)	3 (0.7)	0.497	0 (0)	0 (0)	N.A	0 (0)	2 (1.5)	> 0.999
Pulmonary Cx	2 (3.9)	2 (1.3)	19 (4.3)	0.221	2 (4.8)	0 (0)	0.115	2 (4.2)	6 (4.4)	> 0.999
Neuropsychiatric Cx	0 (0)	0 (0)	3 (0.7)	0.497	0 (0)	0 (0)	N.A	0 (0)	1 (0.7)	> 0.999
** Clavien-Dindo classification, n (%)**				0.045			0.703			0.508
Grade < 3	7 (13.7)	38 (24.7)	108 (24.4)		7 (16.7)	15 (18.5)		7 (14.6)	29 (21.3)	
Grade ≥ 3	5 (9.8)	10 (6.5)	61 (13.8)		5 (11.9)	6 (7.4)		5 (10.4)	17 (12.5)	
Mortality^†^, n (%)	0 (0)	0 (0)	6 (1.4)	0.246	0 (0)	0 (0)	N.A	0 (0)	1 (0.7)	> 0.999

Lap, laparoscopic; EBL, estimated blood loss; WD, well differentiated; MD, moderately differentiated; PD, poorly differentiated; SRC, signet ring cell; RM, resection margin; Cx, complication; N.A., not applicable.

*90-day postoperative morbidity.

^†^90-day postoperative mortality.

**Figure 2:**
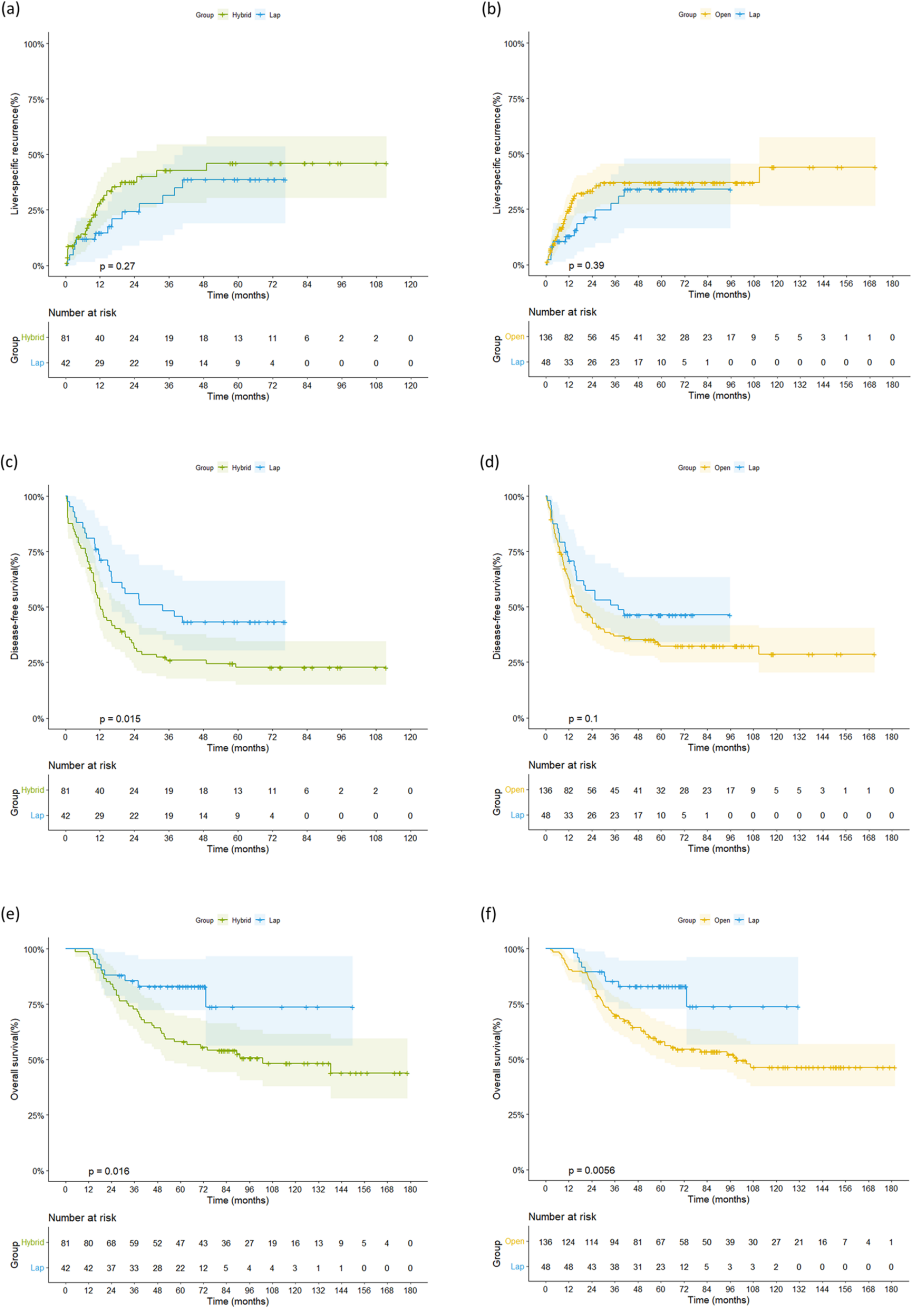
Kaplan–Meier curves of cumulative liver-specific recurrence rate, disease-free survival and overall survival according to treatment group after propensity score matching. The cumulative liver-specific recurrence rates were similar between (**a**) the laparoscopic group and hybrid group, and (**b**) the laparoscopic group and open group. (**c**, **d**) Disease-free survival and (**e**, **f**) overall survival were higher in the laparoscopic groups than in the hybrid group and the open group. Lap, laparoscopic.

Kaplan–Meier curves of the cumulative liver-specific recurrence rate, DFS, and OS for the matched groups are shown in Fig. 2. The respective matched laparoscopic groups showed cumulative liver-specific recurrence rates comparable to the hybrid group (5-year: 38.7% vs. 46.0%, p = 0.270; Fig. 2a) and the open group (34.0% vs. 37.0%, p = 0.391; Fig. 2b). However, the DFS and OS were higher in the laparoscopic group than in the hybrid group (5-year DFS 43.3% vs. 22.7%, p = 0.015, Fig. 2c; 5-year OS 83.0% vs. 58.0%, p = 0.016 Fig. 2e) and the open group (5-year DFS 46.4% vs. 32.2%, p = 0.100, Fig. 2d; 5-year OS 83.0% vs. 57.8%, p = 0.006, Fig. 2f).

## Discussion

In this study, the simultaneous laparoscopic resection of colorectal cancer and liver metastases was safe, feasible, and resulted in adequate oncological outcomes. According to our database comprising three centers, open surgeries were performed in patients with locally more advanced colorectal cancers and multiple, bilobar, and larger liver metastases. After propensity score matching, we found that patients who underwent laparoscopic liver resection recovered better and faster than those who underwent open surgeries, as reflected in a shorter postoperative hospital stay, and lower postoperative wound complication rate. The pathological examination results and clinical outcomes, including the positive resection margin rate of the liver specimens and long-term survival, were comparable between the open and laparoscopic hepatectomy groups.

The simultaneous laparoscopic resection of colorectal cancer and liver metastases is still not widely used, as it requires substantial experience in laparoscopic surgery and a thorough understanding of the intrahepatic vascular and biliary anatomy. Laparoscopic liver resection might increase intraoperative adverse events, such as bleeding due to hepatic vessel injury and inadvertent ligation of vessels and bile ducts. Inadequate resection margins might be another consideration because surgeons cannot palpate the liver tumor with their hands and solely depend on liver MRI and intraoperative ultrasound. In 2017, the Southampton Consensus Guidelines for Laparoscopic Liver Surgery proposed that a laparoscopic simultaneous colonic and liver resection can be considered as a good treatment option in nonrectal primary cancers with peripheral liver lesions^29^. However, there is still insufficient comparative data for combined colorectal and major resections. Furthermore, a cautious expert approach was recommended for metastases involving postero-superior segments or caudate lobe, so called “Difficult Segments (1, 4a, 7, and 8)”, and for metastases close to major vessels, including the hilum, major hepatic veins, and inferior vena cava^30,31^. Despite these concerns, our findings confirm the safety and oncologic adequacy of laparoscopic liver resection. In the laparoscopic group of our study, the rates of postero-superior segments or caudate lobe involvement and close proximity to major vessels of liver metastases were 51.0% and 3.9%, respectively, which were lower than those of the open hepatectomy group. And this might be the cause of lower neoadjuvant chemotherapy of the laparoscopic group with more solitary and unilobar metastases. However, when the variables were adjusted after matching, perioperative outcomes, including operative time, estimated blood loss, and the positive resection margin rates were similar between the laparoscopic and open groups even in the resections of difficult segments. Moreover, there were four cases (7.7%) of conversion to open hepatectomy, and in only one case (1.9%) did this result from major vessel injury. These excellent outcomes may have been facilitated using advanced technology, such as 3D laparoscopy, and new surgical techniques, such as hepatic tumor localization and segmental demarcation using ICG fluorescence.

The comparable short-term outcomes and enhanced postoperative recovery of laparoscopic liver resection compared to open surgery in this study are consistent with those of previous studies^22,23^. Especially, two recent meta-analyses showed that laparoscopic simultaneous resection of colorectal cancer and synchronous liver metastases was associated with less intraoperative blood loss and postoperative complication rate, shorter postoperative hospital stay, and equivalent long-term outcomes than open procedure^32,33^. The reduced blood loss seen in the laparoscopic group may result from more accurate bleeding control under laparoscopic magnification and the hemostatic effect of the pneumoperitoneum^23^. The lower morbidity rate in the laparoscopic group compared to the open group persisted after propensity score matching. This difference mainly resulted from significantly fewer wound complications (laparoscopic group 2.1%; open group 13.2%; p = 0.028). Intestinal anastomotic leakages, which have been theorized to occur more frequently in simultaneous colorectal resection accompanying major liver resection^34,35^ was similar between the groups in this study. Among the liver morbidity, the more biliary collection tendency was observed initially in the laparoscopic group than open hepatectomy groups (5.9% vs. 0.6% vs. 2.9%, p = 0.098), and the difference with the hybrid group was significant after matching. Although the incidence was still low compared to 3.6–33% of previous studies^36^, the higher biliary collection in the laparoscopic group may be due to the pneumoperitoneum, which prevents the bile leak on the cut liver surface during liver parenchymal transection, making it difficult to find the bile leakage points, and the difficulty of controlling the bile leakage points with suture ligation^23,37^.

The hybrid procedure with laparoscopic colorectal resection and open hepatectomy may be an option in institutions where experts with appropriate expertise in both advanced laparoscopic colorectal and liver surgery are not available. Recently, a retrospective study showed that hybrid group had significantly less hemorrhage, shorter postoperative hospital stay, and similar postoperative complications and survivals than those in the open group^38^. However, the surgical outcomes and long-term results of the totally laparoscopic surgery, including major liver resection, compared to hybrid procedures with propensity score matching have not been reported previously. In this study, the surgical outcomes in the hybrid group were analyzed separately from those in the open group and independently compared with those of the laparoscopic group so that the adequacy of the hybrid procedure could be evaluated. The hybrid group initially showed a higher estimated blood loss and overall morbidity rate, and longer postoperative hospital stay compared to those in the laparoscopic group, but the intraoperative blood loss and the postoperative complication rate was not statistically significantly different after matching. The most frequent complication in the hybrid group was urinary retention. Therefore, in situations where laparoscopic liver resection is difficult to perform, the hybrid procedure can be performed with the prospect of morbidity comparable to that of a totally laparoscopic procedure.

In terms of long-term survival, the cumulative liver-specific recurrence rate was similar between the groups in this study. This indicates that laparoscopic liver resection is safe from an oncological perspective. The rapid recovery, reduced morbidity, and shorter hospital stay after laparoscopic surgery had a beneficial effect on oncological outcomes and resulted in better DFS and OS, which, in other studies, have allowed early administration of chemotherapy^39^, better tolerance of repeated hepatectomy for recurrence^40^, and better preservation of immune function^41–43^.

Our results have to be interpreted within the limitations of this study. First, due to the retrospective study design, the possibility of selection bias cannot be excluded. A prospective randomized study would be the ideal study design to compare the surgical outcomes of different procedures. However, such a study is difficult to conduct in this patient population because of the complex selection criteria and the limited number of centers performing simultaneous laparoscopic surgery. Therefore, propensity score matching was the most suitable option we could practically use. Nevertheless, even after matching, there might be an imbalance between the laparoscopic and open hepatectomy groups under the influence of variables that we did not consider. Indeed, in this study, the significantly higher neoadjuvant therapy rate in the hybrid group and the more advanced p/ypT stage tendency in the open group were maintained after matching, which may have resulted in better DFS and OS in the laparoscopic group after propensity score matching. Second, the sample size of the laparoscopic group was relatively small after matching. In previous studies, the laparoscopic groups included from 24 to 109 patients after matching, whereas we included 42 and 48 patients in the two laparoscopic groups^22–25^. However, we analyzed the largest open group, with 442 patients before matching and 136 patients after matching, compared with previous studies. Furthermore, we included a hybrid group (154 patients), which has seldom been assessed to date. Third, the excellent results of this study are from highly experienced tertiary hospitals, performing > 700 laparoscopic colorectal surgery and > 300 laparoscopic liver resections annually, and may not apply to all institutions. Finally, some of the potential advantages of laparoscopic surgery, such as pain scale, recovery time, and quality of life, were not assessed in this study.

In conclusion, simultaneous laparoscopic resection of colorectal cancer and liver metastases in highly experienced centers provides significant short-term benefits, including a shorter postoperative hospital stay and lower postoperative complication rates. In addition, it achieves long-term oncological outcomes comparable with or better than hybrid and open resection.

## Data Availability

The data described in the manuscript is not provided due to privacy and ethical restrictions. However, anonymous data necessary to reproduce the results may be available from the corresponding author on reasonable request.
